# Supplementary material for: A Kolmogorov metric embedding for live cell microscopy signaling patterns
Source: Bioinform Adv. 2025 Mar 22;5(1):vbaf063. doi: 10.1093/bioadv/vbaf063 (PMC12502908; doi:10.1093/bioadv/vbaf063)
Supplement: vbaf063_Supplementary_Data [file vbaf063_supplementary_data.docx]

Supplementary Figure 1: 2-D projections of 3-D (2-D+time) SSF outputs for movies showing ERK signaling in colonies of human induced pluripotent stem cells for 10 differentiated (a) and 10 self-renewing (b) movies. The vertical axis represents the spatial dimension, and is obtained by taking a maximum intensity projection along the second spatial axis. The horizontal axis in each panel represents time. These quantitative visualizations show ERK patterns throughout the process of colony development. The 2-D visualization here is lower dimensional than the 3-D SSF image that is a lossless representation input to the compression distance for embedding. The full dataset can be seen here: https://leverjs.net/ssfCluster/HSC.

Supplementary Figure 2: 2-D projections of 3-D+time ERK (a) and velocity (b) SSF output images for 3-D+time movies of mammary acini, spheroids of human female mammary epithelial MCF10A cells. The original 3-D+time movies are processed with a 3-D SSF via a maximum intensity projection along the *Z* spatial axis to form the input to the compression. For the 2-D rendering shown here, the spatial component is again projected via maximum intensity along the *Y* axis. Each movie is labeled by the time (hours) before optogenetic excitation, the time (hours) that optogenetic excitation lasts (pulses every 30 minutes), and the age of the organoid (days). The dashed vertical lines indicate the beginning and end of the optogenetic excitation. The 2-D projections shown here are useful for human visualization, the 3-D SSF output images are input the FLIF compression algorithm to compute the pairwise NCD to generate the reproducing kernel Hilbert space embedding (5). The optogenetic excitation dataset can be seen here: https://leverjs.net/ssfCluster/optoGenetic.

Supplementary Figure 3: 2-D projections of 3-D SSF output images for 2-D+time movies showing live monolayers of human breast epithelial cells (MCF10A). These 24 movies are from one of six imaging experiments, showing five oncogenic mutations plus wild type (one each per row), also shown as an RKHS embedding in Figure 2 (D). The full dataset contains 147 movies, and can be viewed here: https://leverjs.net/ssfCluster/MCF10A_2D.

Supplementary Movie 1: Animated version of Figure 2. A time-lapse movie showing ERK-KTR signaling in a monolayer of human breast epithelial cells (MCF10A) from the PIK3CA_H1047R mutation with cellular activation indicated by dark nuclei against bright cytoplasm clearly propagating across the image (left panel). The 3-D SSF metric image filter output (center panel) is the input to the FLIF 3-D compression used with the normalized compression distance to define the RKHS embedding, shown here with the current image frame overlaid in gray. The 2-D projection of the 3-D SSF output image (right) panel facilitates human visualization, with the current timepoint indicated by the red line and the signaling patterns clearly visible as diagonal yellow stripes of activation across the monolayer (Link).

Supplementary Movie 2: Rotating view of 3-D SSF output image from Figure 2 (B). The 3-D SSF computes the signaling activation at each cell centroid location $(x,y,time)$. The normalized compression distance finds patterns of similarity between SSF output image pairs. Shown here is an SSF output image for the PIK3CA_H1047R oncogenic mutation known for distinct ERK signaling patterns, (Link).
